# Toward a global DNA barcode reference library of the intolerant nonbiting midge genus *Rheocricotopus* Brundin, 1956

**DOI:** 10.1002/ece3.7979

**Published:** 2021-08-04

**Authors:** Xiao‐Long Lin, Kun Jiang, Wen‐Bin Liu, Wei Liu, Wen‐Jun Bu, Xin‐Hua Wang, Lidong Mo

**Affiliations:** ^1^ College of Life Sciences Nankai University Tianjin China; ^2^ Tianjin Key Laboratory of Conservation and Utilization of Animal Diversity Tianjin Normal University Tianjin China; ^3^ Crowther Lab Institute of Integrative Biology ETH Zurich (Swiss Federal Institute of Technology) Zurich Switzerland

**Keywords:** bioindicators, cryptic species, DNA barcode, environmental factors, species delimitation

## Abstract

Environmental DNA metabarcoding is becoming a predominant tool in biodiversity assessment, as this time‐ and cost‐efficient tactics have the ability to increase monitoring accuracy. As a worldwide distributed genus, *Rheocricotopus* Brundin, 1956 still does not possess a complete and comprehensive global DNA barcode reference library for biodiversity monitoring. In the present study, we compiled a cytochrome *c* oxidase subunit 1 (COI) DNA barcode library of *Rheocricotopus* with 434 barcodes around the world, including 121 newly generated DNA barcodes of 32 morphospecies and 313 public barcodes. Automatic Barcode Gap Discovery (ABGD) was applied on the 434 COI barcodes to provide a comparison between the operational taxonomic units (OTU) number calculated from the Barcode Index Number (BIN) with the “Barcode Gap Analysis” and neighbor‐joining (NJ) tree analysis. Consequently, these 434 COI barcodes were clustered into 78 BINs, including 42 new BINs. ABGD yielded 51 OTUs with a prior intraspecific divergence of *Pmax* = 7.17%, while NJ tree revealed 52 well‐separated clades. Conservatively, 14 unknown species and one potential synonym were uncovered with reference to COI DNA barcodes. Besides, based on our ecological analysis, we discovered that annual mean temperature and annual precipitation could be considered as key factors associated with distribution of certain members from this genus. Our global DNA barcode reference library of *Rheocricotopus* provides one fundamental database for accurate species delimitation in Chironomidae taxonomy and facilitates the biodiversity monitoring of aquatic biota.

## Graphical abstract

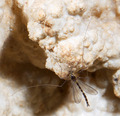

## 1 INTRODUCTION

Biodiversity has been declining under the pressure from nonstopping human activities coupled with ongoing climate change events (Newbold et al., 2015) and needs urgent protective actions. However, a thorough understanding of biodiversity with its temporal dynamics (Turner, 2014) is the prerequisite for the effective biodiversity conservation. Traditionally, biodiversity monitoring in freshwater ecosystems is particularly dependent on taxonomic expertise with classical morphological information (Kelly et al., 2014). To overcome this limitation, DNA barcoding (Hebert et al., 2003; Hebert et al., 2003) has gradually and widely been applied on species identification as well as taxonomic assessments with the support of standardized genetic markers (Hebert et al., 2004; Young et al., 2019). Thanks to the advances in DNA sequencing technologies, DNA metabarcoding (Tab erlet et al., 2012; Yu et al., 2012) based on organismal and/or environmental DNA (eDNA) has become increasingly popular in facilitating biodiversity assessment and biomonitoring freshwater biota (e.g., Carew et al., 2013; Elbrecht et al., 2017; Ficetola et al., 2008; Sun et al., 2019; Tab erlet et al., 2018; Valentini et al., 2016). A comparison is invited between traditional morphology‐based biomonitoring approaches and eDNA metabarcoding which is capable of providing increased monitoring accuracy together with economical and time efficiency; thus, this technique serves as a valuable research tool for biodiversity monitoring and environmental policy making (Kelly et al., 2014). However, eDNA‐based biodiversity assessments of freshwater ecosystem are still highly limited with only a narrow assortment of freshwater macroinvertebrate DNA barcode reference libraries currently available, such as caddisflies, chironomids, mayflies, and stoneflies (Carew et al., 2017; Galimberti et al., 2021; Leese et al., 2018; Morinière et al., 2017, 2019). The abovementioned libraries are just a tip of the iceberg of freshwater macroinvertebrate species diversity.

Chironomids (Diptera: Chironomidae) have the most abundant species‐rich genera among benthic invertebrates (Armitage et al., 1995), comprising of more than 6,300 accepted species (P. Ashe, personnel communication) in all zoogeographical regions, even in areas with an extreme environment like Antarctica (Rico & Quesada, 2013). As a major component of biodiversity, chironomids are not only valuable sources for phylogenetic and biogeographical research (Brundin, 1966; Cranston et al., 2012; Krosch & Cranston, 2013; Lin et al., 2018c), but also act as important bioindicators for freshwater ecosystems monitoring (Ferrington, 2008). The theoretical species number of Chironomidae is likely to exceed 20,000 (Armitage et al., 1995), which introduces the difficulty to identify species by traditional morphological approaches. Since DNA barcoding is able to provide the chance to perform accurate and comprehensive species identifications, this effective strategy is urgently needed as a steppingstone to facilitate evolutionary studies and biodiversity assessments.

**FIGURE 1:**
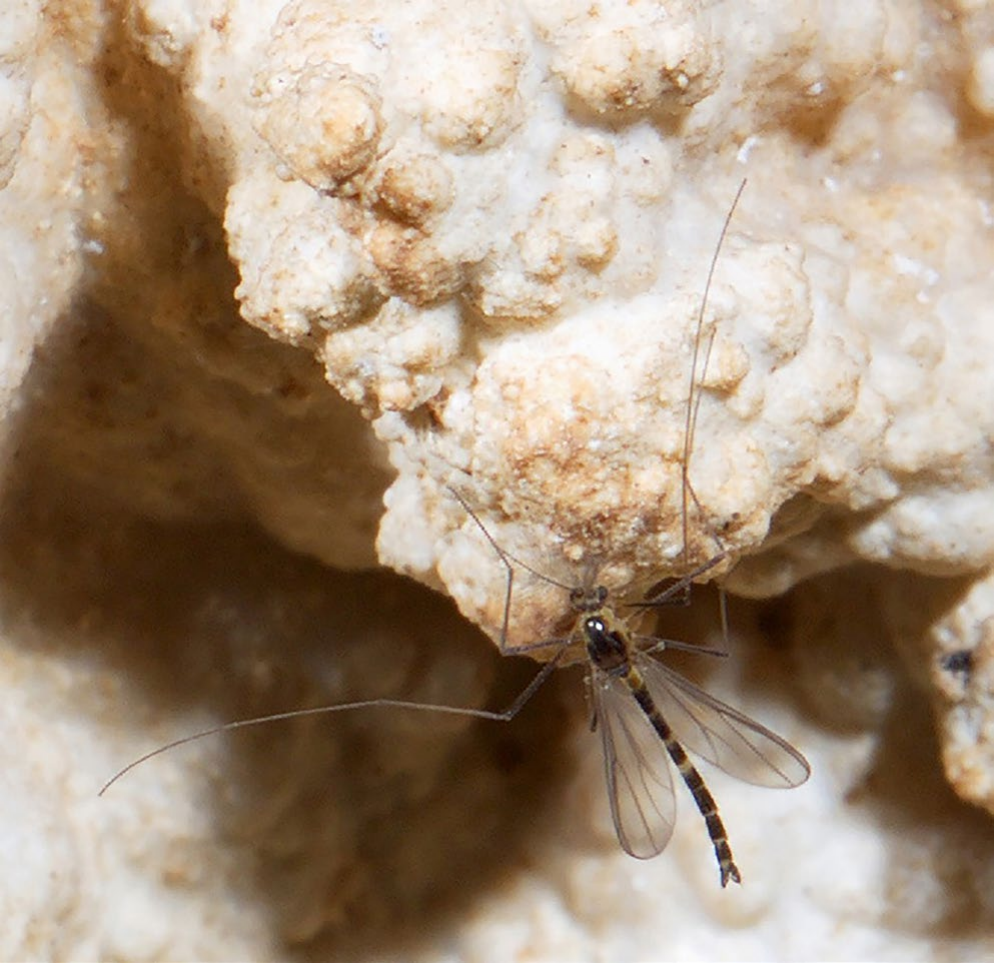
An unknown *Rheocricotopus* species found in a gypsum karst cave in Guizhou Province, China. Photo courtesy: Mr. Wei‐Wei Zhang in 2020

**FIGURE 2:**
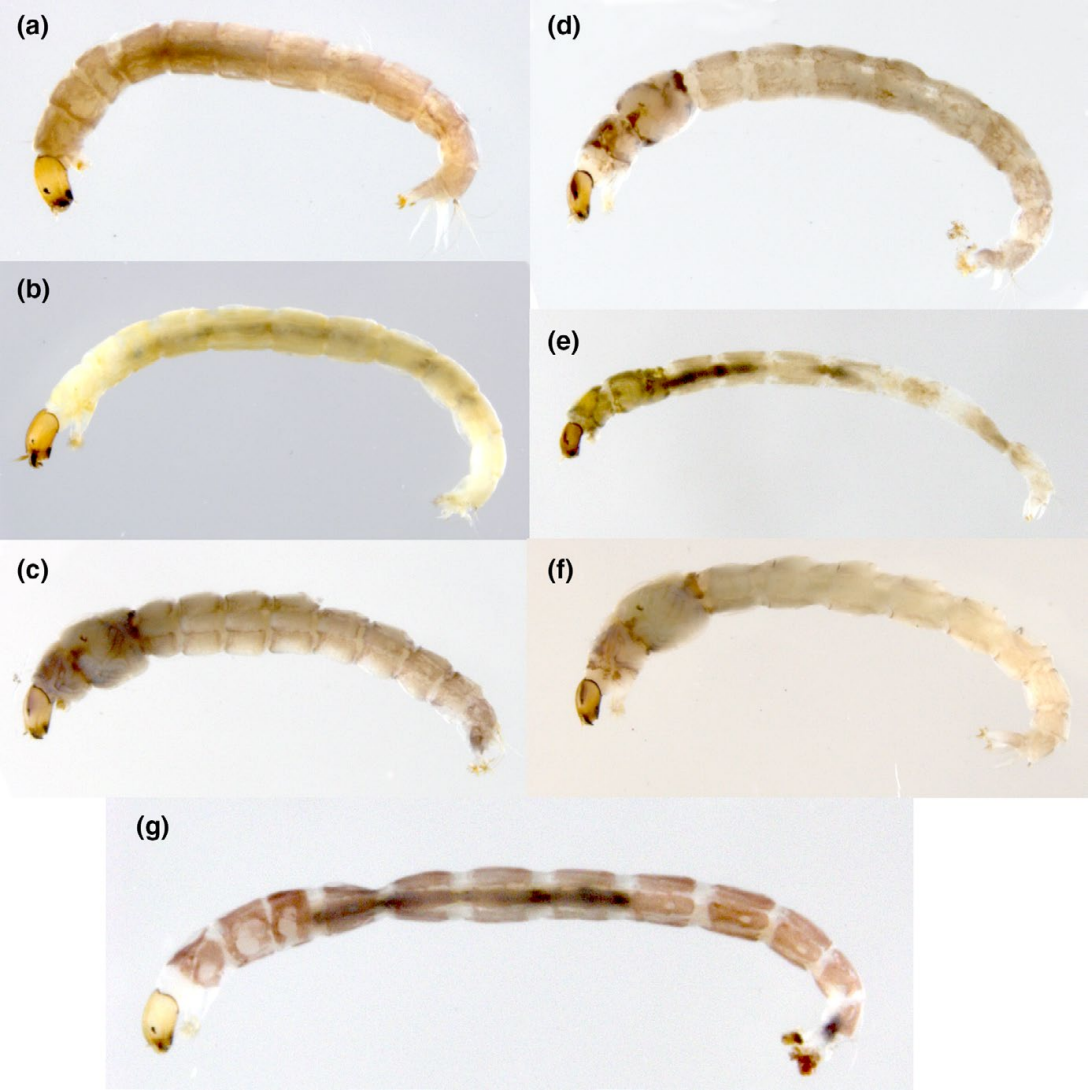
*Rheocricotopus* larvae in 95% ethanol. (a) *Rheocricotopus brachypus* Wang & Zheng, 1991; (b) *Rheocricotopus brochus* Liu et al., 2014; (c) *Rheocricotopus chalybeatus* (Edwards, 1929); (d) *Rheocricotopus emeimensis* Wang & Zheng, 1989; (e) *Rheocricotopus taiwanensis* Wang, Yan & Maa, 2004; (f) *Rheocricotopus tamahumeralis* Sasa, 1981; g. *Rheocricotopus* sp. 15XL

*Rheocricotopus* Brundin, 1956 (Figure 1) is a species‐diverse genus of subfamily Orthocladiinae, family Chironomidae with ca. 80 valid identified species worldwide so far (Ashe & O'Connor, 2012; Lin et al., 2020; Moubayed‐Breil & Ashe, 2019). The larvae of *Rheocricotopus* species (Figure 2) mainly inhabit the lotic water (i.e., streams and rivers in alpine mountains), whereas a few species occur in freshwater lakes. Hence, the intolerant species of *Rheocricotopus* are regarded as one of the most important bioindicators for freshwater ecosystem monitoring. The genus *Rheocricotopus* was erected based on the type species *Rheocricotopus effusus* (Walker, 1856) by Brundin (1956) and since, a wide array of species groups and species of *Rheocricotopus* all around the world have been revised over the past decades (Liu et al., 2014; Liu et al., 2014; Sæther, 1985). However, some remaining challenges still worth further consideration in the process of taxonomic disentangling of *Rheocricotopus*. For example, information derived from poorly investigated regions is scarce (e.g., Australian and Oriental regions) and data of incomplete life stages could be considered as another major obstacle due to the fact that investigators always have trouble matching immature individuals with adults reared in the field. Besides, ambiguous boundaries do exist among those closely related species from a morphological perspective. Classically, morphological taxonomy to species level strongly relies on the traits of adult males of *Rheocricotopus*. For instance, some key diagnostic characters, including coloration, wing setation and shape of hypopygium, could contribute to morphological identification, but far from adequate to successfully distinguish a narrow range of intraspecific variations.

DNA barcodes could provide a more precise and effective approach to disentangle biodiversity in genus *Rheocricotopus*. Although a few public DNA barcodes of *Rheocricotopus* have been published in previous studies (e.g., Lin et al., 2020), the global DNA barcode reference library still remains incomplete. In this study, by compiling cytochrome *c* oxidase subunit 1 (COI) DNA barcodes of 434 individuals, we aim to uncover unknown life stages and cryptic species and clarify species boundaries of closely related species of *Rheocricotopus*. Additionally, a thorough understanding concerning ecological characteristics of this globally distributed genus is the final goal for providing clues to exploring the potential relationship between distribution and environmental factors.

## 2 MATERIALS AND METHODS

### 2.1 Taxon sampling and identification

**FIGURE 3:**
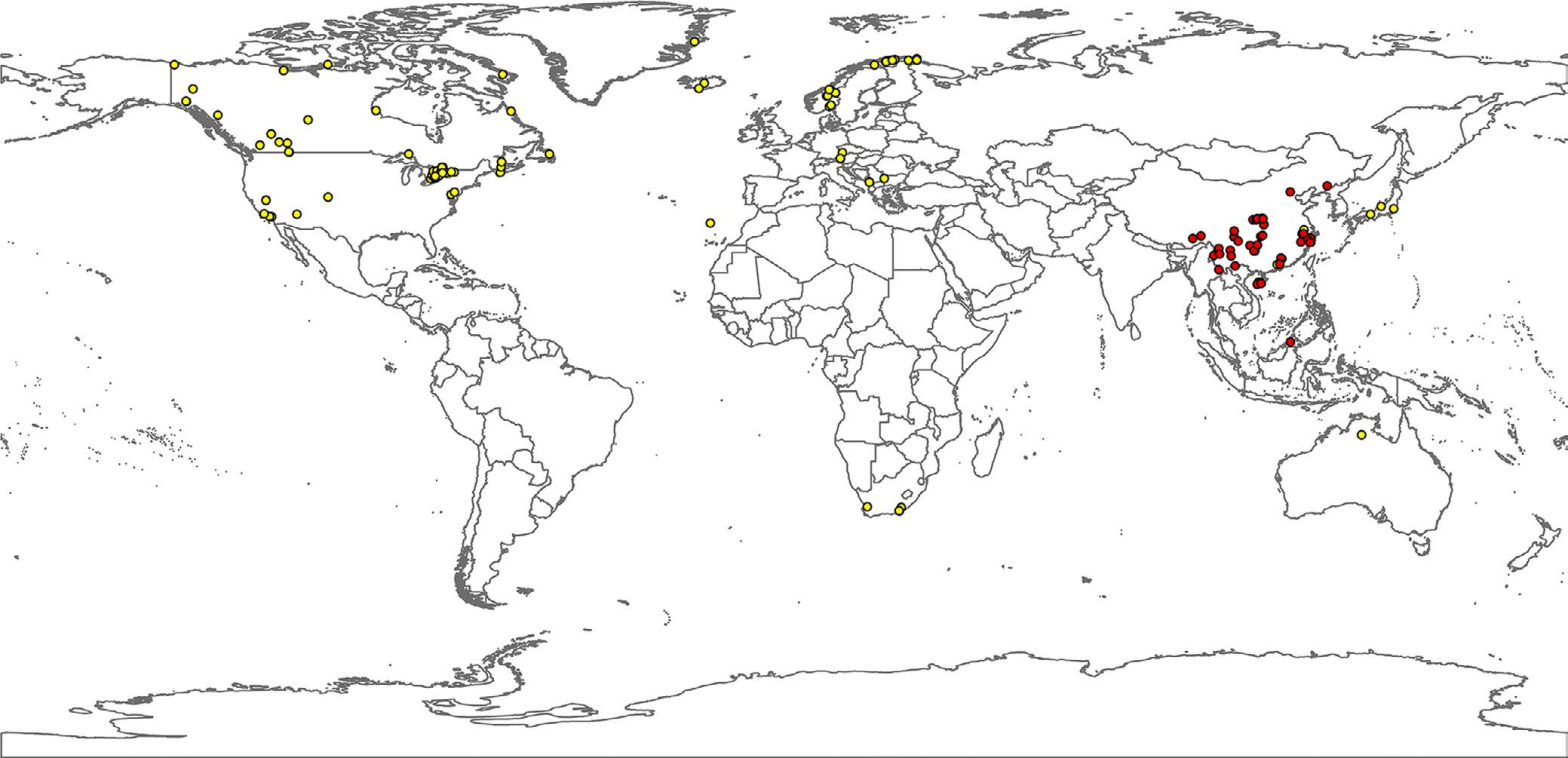
Distribution map of 434 individuals of the *Rheocricotopus*. Red dots represent new records from this study

Fieldwork was conducted in China and Malaysia during 2008 to 2020 (Figure 3), and 121 specimens of *Rheocricotopus* were collected. Adult specimens were collected mainly using sweep net and malaise trap and preserved in 85% ethanol. Immature specimens were collected using drift net and D‐net and preserved in 95% ethanol at dark. Specimens were identified using taxonomic revisions and species descriptions (Lin et al., 2020; Liu et al., 2014; Liu et al., 2014; Sæther, 1985; Wang, 1995; Wang & Sæther, 2001; Wang & Zheng, 1989, 1991). The voucher specimens are deposited at the College of Life Sciences, Nankai University.

### 2.2 Molecular laboratory work

Genomic DNA of most specimens was extracted from head‐thorax using Qiagen DNA Blood and Tissue Kit according to the manufacture's instruction. PCR amplifications of COI barcodes with the universal primers LCO1490 and HCO2198 (Folmer et al., 1994) were performed following the protocol from Lin et al. (2018a). Sanger sequencing of the purified PCR products was carried on the ABI 3730 at the BGI (Beijing, China). In addition, genomic DNA extraction from three legs, PCR amplification, and high‐throughput sequencing of the specimens were conducted at the Canadian Centre for DNA Barcoding (CCDB, University of Guelph, Canada) using standard high‐throughput protocols (deWaard et al., 2008; Hebert et al., 2018). DNA samples are deposited at the College of Life Sciences, Nankai University, Tianjin, China, and the CCDB.

### 2.3 DNA barcodes analysis

Raw sequences were edited and assembled in Geneious Prime version 2021.0.3 and aligned using MUSCLE (Edgar, 2004) implemented in MEGA X (Kumar et al., 2018) to check stop codons.

To obtain DNA barcodes, we searched for public COI barcodes of *Rheocricotopus* that were longer than 400 base pairs with a lack of stop codons in the Barcode of Life Data System (BOLD, http://www.boldsystems.org/) (Ratnasingham & Hebert, 2007). In total, a dataset “Global DNA barcodes of the genus *Rheocricotopus* (DS‐2020RHEO)” including 434 COI barcodes of *Rheocricotopus* were correspondingly generated on BOLD (3 December 2020), of which 121 COI barcodes representing 32 species were originated from this study, while the remaining 313 sequences of 23 species were publicly acquired from BOLD and GenBank. Three species were overlapping between the new DNA barcodes and the published ones.

Firstly, all 434 COI barcode sequences were applied to the Barcode Index Number (BIN) system on BOLD (Ratnasingham & Hebert, 2013). The BIN system clusters DNA barcodes to generate OTUs with a threshold of 2.2% for a rough differentiation between interspecific and intraspecific genetic distances (Ratnasingham & Hebert, 2013). In addition, we used the “Barcode Gap Analysis” tool on BOLD to calculate sequence divergences for the present dataset, for example, the mean and maximum pairwise distances for intraspecific divergences, the mean and minimum pairwise distances for interspecific divergences, and minimum genetic distances to the nearest neighbor.

Moreover, a neighbor‐joining (NJ, Saitou & Nei, 1987) tree was constructed based on the 434 COI barcodes using the Kimura 2‐Parameter (K2P) model (Kimura, 1980) with 1,000 nonparametric bootstrap replicates and pairwise deletion in MEGA X.

Given that Automatic Barcode Gap Discovery (ABGD) provides a more reliable approach for species delimitation based on the single‐locus marker (Lin et al., 2018b; Pentinsaari et al., 2017), our data were applied into the ABGD to compare the OTU number resulting from the BIN‐based “Barcode Gap Analysis” with the constructed NJ tree. ABGD analysis was carried out on 17 January 2021 using the web interface (https://bioinfo.mnhn.fr/abi/public/abgd/abgdweb.html). We used the K2P model, *Pmin* = 0.005 and kept default settings for remaining parameters.

Finally, a haplotype network for COI barcodes of a potential cryptic species complex was reconstructed with PopART (Leigh & Bryant, 2015) using the TCS method (Clement et al., 2000, 2002) with gaps and missing data excluded.

### 2.4 Ecological analysis

To explore the environmental factors that could possibly determine the distribution of the genus *Rheocricotopus*, certain corresponding environmental factors were extracted for each sampling location These environmental factors included 19 bioclimatic variables (Karger et al., 2017), frost days and frequency (Karger et al., 2017), aridity index (Trabucco & Zomer, 2010), cloud cover (Wilson & Jetz, 2016), the global habitat heterogeneity information (Tuanmu & Jetz, 2015), topographic information of elevation and slope (Robinson et al., 2014), snow probability (Hall et al., 2006), depth to water table (Fan et al., 2013), Hansen tree cover 2010 (Hansen et al., 2013), population density (Center for International Earth Science Information Network, 2018), vegetation index (NDVI) (Didan et al., 2015), and productivity (Running et al., 2011). Afterward, we applied principal component analysis on these environmental factors and presented the clustering and distribution pattern on the top two principal component analysis (PCA) dimensions. Furthermore, to understand the niche diversification of the most prominent environmental variables, we presented the density distribution of different distribution groups with temperature and precipitation gradients. Specifically, the samples are grouped as following: east and southeast Asia (EA), Europe (EU), North America (NAC), and Africa (AF).

## 3 RESULTS

### 3.1 DNA barcode analysis

**FIGURE 4:**
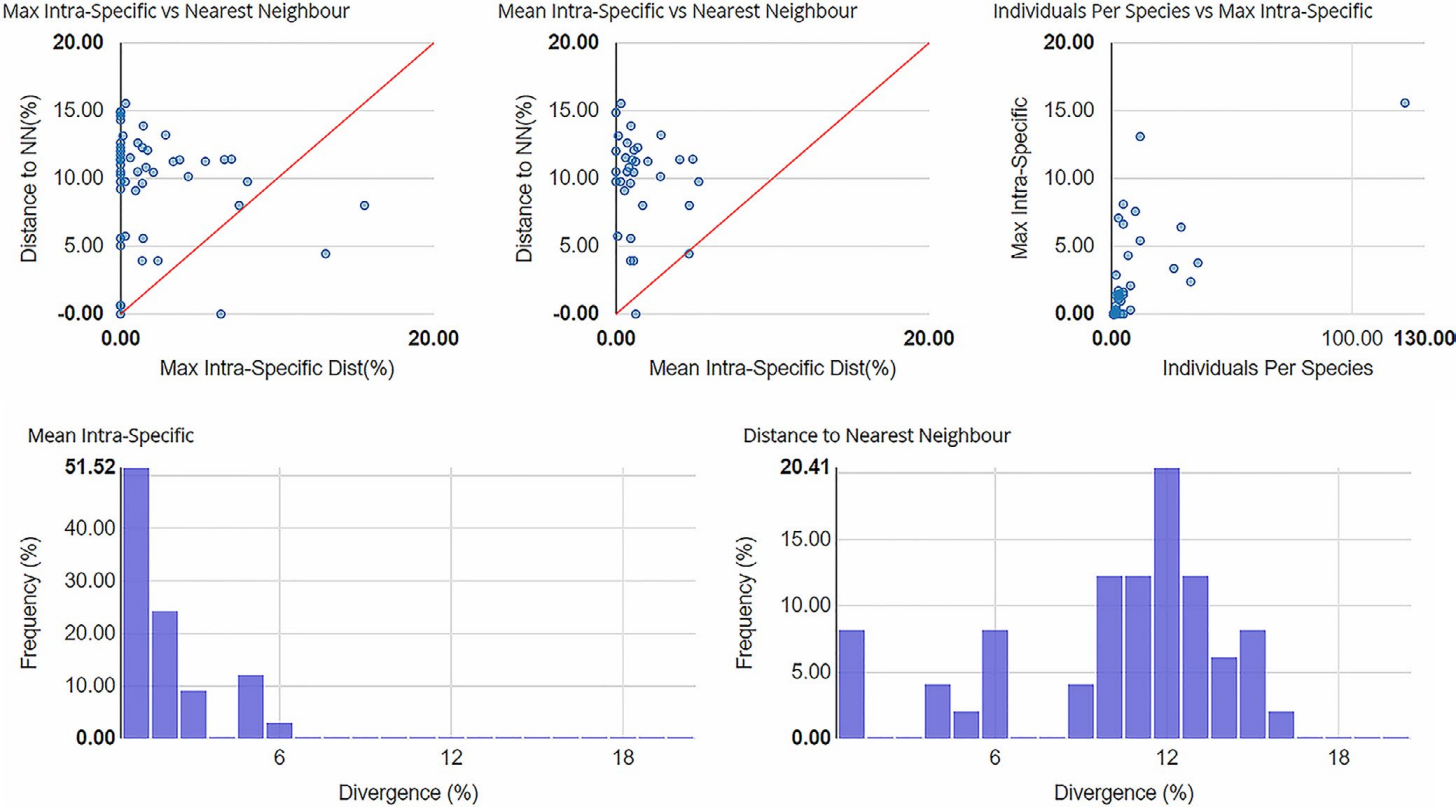
Barcode Gap Analysis of 434 COI barcode sequences of 52 *Rheocricotopus* species. Two distance distribution histograms show the mean intraspecific divergence and distances to nearest neighbor. Three scatter plots are provided to confirm the existence and magnitude of the Barcode Gap. The first two scatter plots show the overlap of the max and mean intraspecific distances versus the interspecific (nearest neighbor) distances. The third scatter plot shows the number of individuals in each species against their max intraspecific distances, as a test for sampling bias

The aligned 434 COI sequences ranged from 407 to 658 base pairs, including 132 sequences with a full barcode length of 658 base pairs. These 434 sequences were assigned to 78 BINs, including 50 concordant BINs, 26 singleton BINs, and 2 discordant BINs. 42 new BINs were added to BOLD. The following 13 species were represented by at least 2 BINs: *Rheocricotopus atripes* (Kieffer, 1913), *Rheocricotopus brachypus* Wang & Zheng, 1991, *Rheocricotopus chalybeatus* (Edwards, 1929), *Rheocricotopus chapmani* (Edwards, 1935), *Rheocricotopus effusus* (Walker, 1856), *Rheocricotopus emeimensis* Wang, 1991, *Rheocricotopus fuscipes* (Kieffer, 1909), *Rheocricotopus nigrus*, Wang & Zheng, 1989, *Rheocricotopus robacki*, (Beck & Beck, 1964), *Rheocricotopus* sp. 3XL, *Rheocricotopus taiwanensis* Wang, Yan & Maa, 2004, *Rheocricotopus tibialis* Wang & Zheng, 1991, and *Rheocricotopus valgus* Chaudhuri & Sinharay, 1983. The mean intraspecific divergence of all species was 1.51%, while the mean interspecific divergence was 14.78% (Figure 4).

### 3.2 Species discrimination

In general, our results showed a consistent matching pattern between molecular OTU and morphospecies in *Rheocricotopus*. The NJ tree included 52 well‐separated clusters representing 25 named, 19 unnamed and eight unidentified morphospecies. After examination of accessible unnamed species, we concluded that 14 species might be new to science whereas the remaining specimens were deemed as unidentified. *Rheocricotopus brochus* Liu et al., 2014 could probably be a junior synonym of *Rheocricotopus bifasciatus* Wang & Zheng, 1991 with the evidence of low interspecific genetic distance (File S1).

### 3.3 Species without identification

Of the 313 sequences obtained from public COI barcode, only a few species are identified at the genus level because we do not have access to the vouchers for morphological examination. Besides, a number of species could not be morphologically identified to species level since the immatures and adult females of *Rheocricotopus* have not been described.

### 3.4 Cryptic species diversity

**FIGURE 5:**
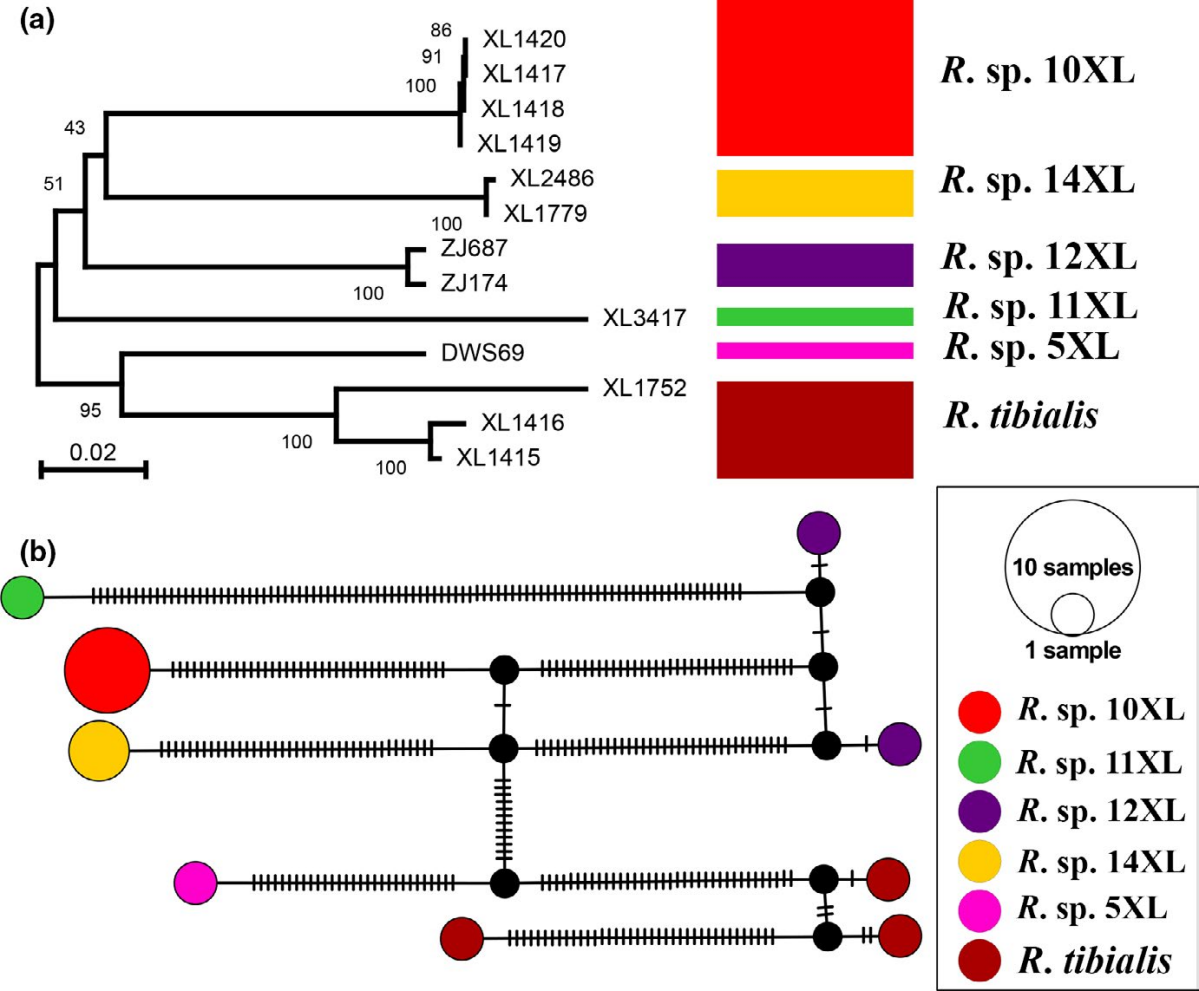
Genetic analyses of 13 COI barcodes of the *Rheocricotopus tibialis* species complex. (a) Neighbor joining tree of *R*. *tibialis* species complex based on K2P distance; numbers on branches represent bootstrap based on 1,000 replicates; scale represents K2P genetic distances. (b) TCS haplotype network based the COI barcodes of the *R*. *tibialis* species complex. Mutations are shown as lines on the branches

Based on our results, DNA barcodes show great cryptic species diversity within the *Rheocricotopus chalybeatus* species group (File S1). For instance, both NJ tree and TCS haplotype network based on 13 DNA barcode sequences of the *Rheocricotopus tibialis* species complex revealed six OTUs (Figure 5). Five putative cryptic species (*Rheocricotopus* sp. 5XL, *Rheocricotopus* sp. 10XL, *Rheocricotopus* sp. 11XL, *Rheocricotopus* sp. 12XL, and *Rheocricotopus* sp. 14XL) are closely related to *Rheocricotopus tibialis* Wang & Zheng, 1991 with highly similar hypopygia and tergite coloration of adult males. These cryptic species could be differentiated from others within the species complex by multiple nuclear markers (Lin, unpublished) and morphological characters. Additionally, similar cases could be found in *Rheocricotopus atripes*, *Rheocricotopus chalybeatus,* and *Rheocricotopus robacki* species complexes (File S1).

### 3.5 Life stage association

Our data provided proof that the larvae of seven species (Figure 2) and adult females of nine species from China were associated with their adult males with the aid of DNA barcodes (File S1). Among above cases, the larvae of *Rheocricotopus brachypus* Wang & Zheng, 1991, *R*. *brochus*, *R*. *emeimensis*, *Rheocricotopus tamahumeralis* Sasa, 1981, and *R*. *taiwanensis* have not been described yet. In addition, adult females of *Rheocricotopus calviculus*, Wang & Sæther, 2001, *R*. *emeimensis*, *Rheocricotopus godavarius* Lehmann, 1969, *Rheocricotopus inaxeyeus* Sasa, Kitami & Suzuki, 2001, *R*. *nigrus,* and *Rheocricotopus tibialis*, were reported for the first time in this study.

### 3.6 OTU delineation based on DNA barcodes using ABGD

**FIGURE 6:**
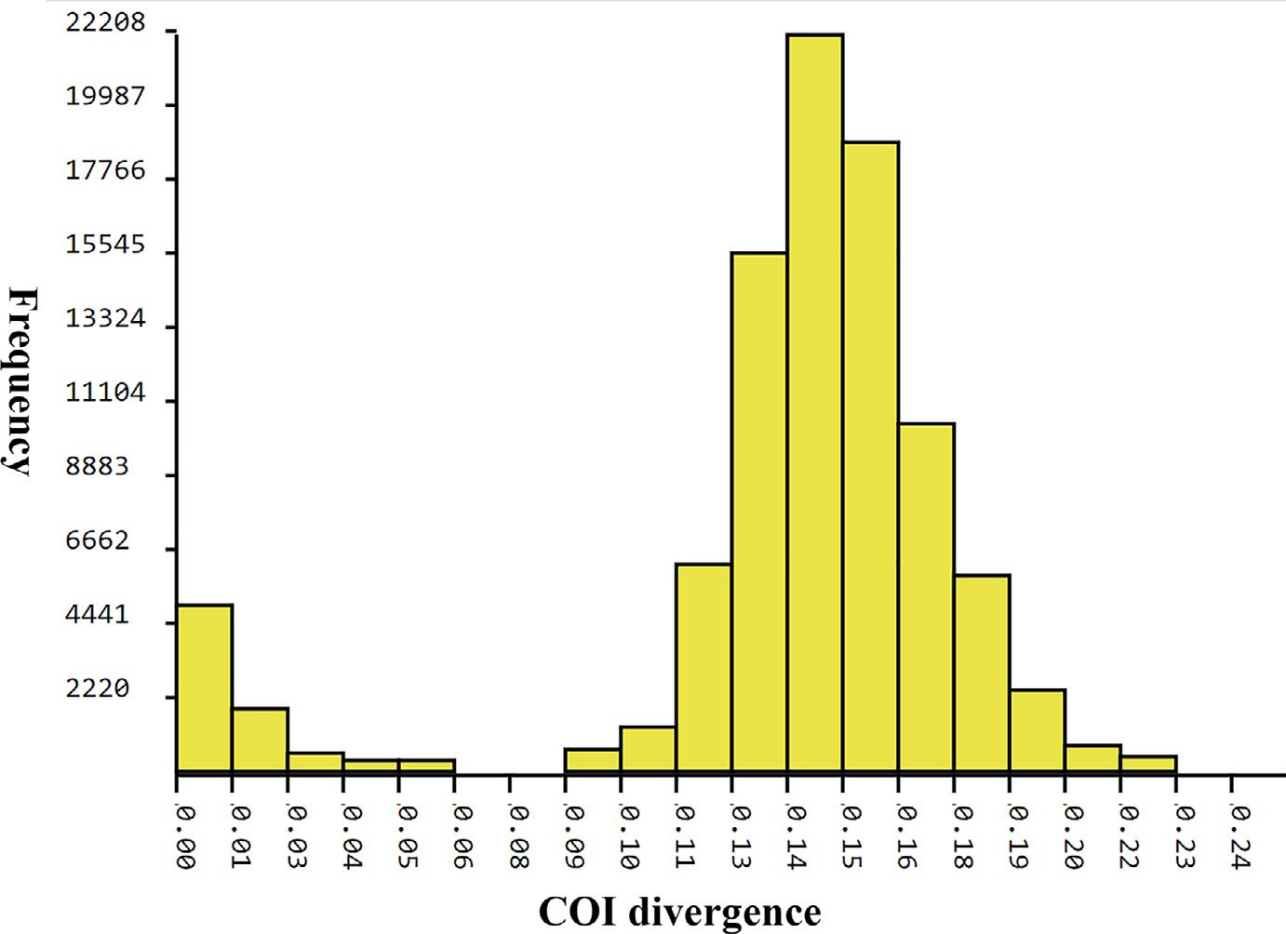
Histogram of pairwise K2P distances of 434 COI barcodes of *Rheocricotopus*

A small “barcode gap” was observed on pairwise distance (Figure 6). ABGD analysis of the present dataset recognized 51 OTUs with a prior intraspecific divergence of *Pmax* = 7.17%.

### 3.7 Ecological analysis results

**FIGURE 7:**
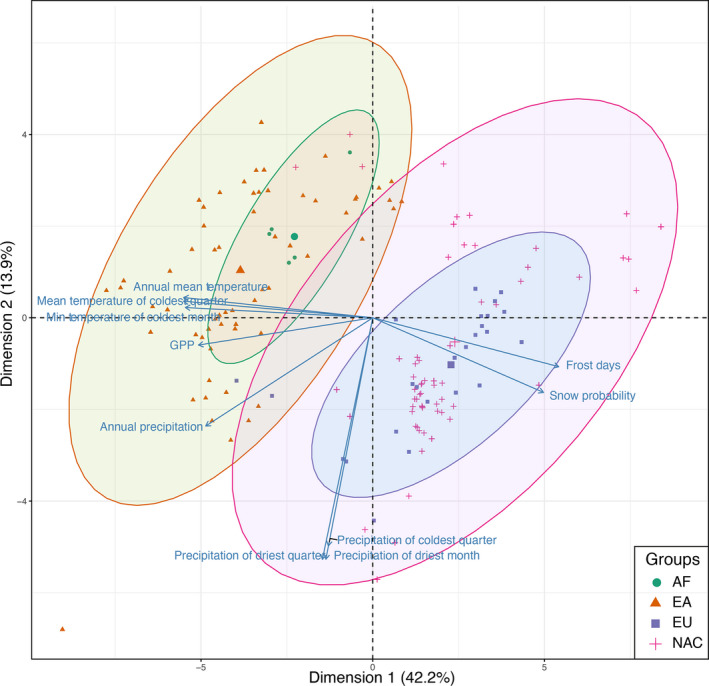
Distribution of the four species groups from *Rheocricotopus* in the 2‐dimension space of the top two dimension from PCA analysis. Dimension 1 and dimension 2 represent the variation 42.2% and 13.9%, respectively. Top 10 important variables presented here are mainly temperature‐ and precipitation‐associated variables. Specifically, temperature‐associated variables dominate dimension 1, while precipitation‐associated variables dominate dimension 2. The four groups east and southeast Asia (EA), European group (EU), North America (NAC), and Africa (AF) are displayed based on their spatial locations in 2‐dimension space of dimension 1 and 2

The first two axes of PCA (Figure 7) explained 42.2% and 13.9% of the variation, respectively, showing spatial grouping of certain environmental variables. Specifically, dimension 1 represented the temperature gradient with annual mean temperature and frost days *etc*., while dimension 2 represented the precipitation gradient. The Asia group (EA) and North America (NAC) group were separated into two independent clusters, while the two groups from Africa and Europe nested inside the EA and NAC group, respectively. The top 10 important variables were temperature and precipitation, and their derivative variables highly correlated (Pearson's correlation coefficients varied from 0.62 to 1) with annual mean temperature or annual precipitation (File S2, File S3). Furthermore, the density plots of temperature and precipitation showed similarities and diversification of EU, NAC, EA, and AF groups. EA group has a wider niche range on precipitation, while the other three groups have narrow range with drier environmental conditions. EA and AF have similar temperature niche range and are diversified with EU and NAC groups.

## 4 DISCUSSION

### 4.1 Global DNA barcode reference library of intolerant *Rheocricotopus* species

Since *Rheocricotopus* nonbiting midges are intolerant to potential pollutants, appropriate identifications are crucial for biomonitoring implemented in the conservation and management of freshwater ecology. In this study, we investigated the taxonomy of Chinese and Malaysian *Rheocricotopus* nonbiting midges and hence made contribution to the global DNA barcode reference library. Currently, the formed‐library includes 434 records for 78 BINs representing 52 putative species. Until now, 25 species have Linnaean names and the remaining 27 species do not possess Linnaean names with 14 species new to science and 13 unidentified specimens. Overall, DNA barcodes coverage is more than 50% for known *Rheocricotopus* species. Our results also suggested a rich cryptic species diversity, indicating that the number of species within *Rheocricotopus* is likely to be much higher than previously recognized. Particularly, 13 species from China and one species from Malaysia could be new to science. As an environmental sensitive genus with narrow distribution range, specimens of *Rheocricotopus* are hard to be sampled in field. With the limitation of research funding and close collaborator research focus, the main field sampling work was conducted in East Asia. The DNA barcodes of *Rheocricotopus* from EU, AM, and AF were acquired from open database, which is the maximum recordings we can accumulate up to now. At last, as a small genus with less attention, data access and sharing are facing big challenges. Therefore, wider sampling of *Rheocricotopus* is still needed for building a comprehensive DNA barcode reference library. Compared with classical morphological approaches, biodiversity assessments of freshwater ecosystems based on DNA metabarcoding are time‐saving and cost‐efficient tactics. Nevertheless, the establishment of DNA barcode libraries for macroinvertebrates remain scarce, particularly in some developing counties with relatively rich biodiversity. The present study is a significant contribution to build a more comprehensive DNA barcode library for macroinvertebrates.

### 4.2 OTU and taxonomy

Over the last decades, DNA barcodes have been increasingly utilized in chironomid species discovery and identifications (Anderson et al., 2013; Carew et al., 2007; Ekrem et al., 2010; Lin et al., 2015, 2018a, 2019, 2020; Silva & Wiedenbrug, 2014; Song et al., 2018). In this study, we demonstrated that DNA barcoding can serve as an efficient tool for species delimitation and life stage association within *Rheocricotopus*. A number of larvae of some species are reported for the first time as putative new species with the assistance of DNA barcodes. In general, ABGD and NJ tree based on COI DNA barcodes yield concordant OTUs corresponding to morphospecies. Undoubtedly, deep intraspecific divergence on COI barcode can lead to overestimation of the species diversity. Moreover, insufficient taxon sampling (Luo et al., 2015), incomplete lineage sorting (Pollard et al., 2006; Willyard et al., 2009) and horizontal gene flow (Polz et al., 2013) can lead to incorrect species delimitation. Therefore, a further integrative taxonomy with reference to both morphology and molecules is required to sort out species boundaries of those closely related species.

Since different species have distinct population size and divergence time, a universal and fixed threshold is not appropriate for all macroinvertebrates (Yang & Rannala, 2017). Initially, Hebert et al. (2004) proposed “10X rule”, meaning interspecific divergence at least 10 times as large as the intraspecific divergence. As a result, low threshold of 2%–3% was suggested to offer effective identification to the species level for some groups of Coleoptera, Diptera, Heteroptera, Lepidoptera, Plecoptera, and Trichoptera (Knebelsberger et al., 2014; Monaghan et al., 2005; Schmidt et al., 2015; Zahiri et al., 2014; Zhou et al., ,^2010^, ^2016^). However, higher thresholds could be found in some macroinvertebrate groups along with increased sampling size. For instance, Hydropsychidae holds the threshold of 6%–8% (Pauls et al., 2010). According to recent studies with regard to chironomid DNA barcodes (Carew & Hoffmann, 2015; Lin et al., 2015, 2018b; Song et al., 2018), a higher threshold of 3%–8% is proper for Chironomidae. Despite of the cryptic species, the maximum intraspecific divergence of Chironomidae is even up to 10% found in *Tanytarsus thomasi* Lin, Stur *et* Ekrem, 2018 (Lin et al., 2018a). Therefore, there is a challenge for eDNA metabarcoding using a threshold of 3% to separate OTUs for some taxonomic groups. To overcome this challenge, more diverse geographic populations per species should be barcoded to strengthen a more comprehensive and reliable database for the species annotation for eDNA metabarcodes.

### 4.3 Environmental determination of *Rheocricotopus* distribution

The importance of temperature for the distribution of Chironomids discovered in this work is consistent with previously published studies (Medeiros et al., 2021; Medeiros & Quinlan, 2011). Notably, precipitation is proved to be associated with the distribution of chironomids, which has not been mentioned before. However, other environmental parameters, such as water temperature and pH, are necessarily needed for investigators to further disentangle the evolution and adaptation of *Rheocricotopus*. The clustering pattern (EU and NAC; AF and EA) achieved from PCA analysis illustrated a possible adaptation diversification of the EU and NAC groups to colder environmental conditions while that of AF and EA groups to warmer habitats. As north America and European continents were heavily affected by the late quaternary climate oscillations with repeating land ice sheet advancing and retreating (Batchelor et al., 2019; Svendsen et al., 2004), driving the diversification of the *Rheocricotopus* species toward colder habitats. Species from south Africa, East and southeast Asia were exempted from these strong impacts. Additionally, phylogenetic studies (Ekrem et al., 2018) also argued some potential genetic communications among sister genera of *Rheocricotopus*. However, we need further investigations from the field and studies to disentangle the evolution and speciation history of *Rheocricotopus* or chironomids.

## 5 CONCLUSION

Our results demonstrated that important role of DNA barcodes in the discovery of cryptic species and association of life stages of intolerant *Rheocricotopus* nonbiting midges. The global DNA barcode reference library of *Rheocricotopus* now includes 434 records for 78 BINs representing 52 putative species, contributing to accurate species delimitation in Chironomidae taxonomy and the monitoring of aquatic biota. Besides, we showed that the distributions of *Rheocricotopus* nonbiting midges are mainly associated with temperature and precipitation. Meanwhile, the similarities of EU and NAC groups provided another potential evidence for the possibility that historical climate dynamics could probably determine the present species distribution and adaptation to environment.

## CONFLICT OF INTEREST

The authors declare that they have no conflicts of interest.

## AUTHOR CONTRIBUTIONS

**Xiao‐Long Lin:** Conceptualization (equal); data curation (lead); formal analysis (lead); funding acquisition (equal); investigation (lead); methodology (equal); project administration (lead); resources (equal); supervision (equal); writing‐original draft (equal); writing‐review and editing (equal). **Kun Jiang:** Formal analysis (equal); writing‐original draft (equal). **Wen‐Bin Liu:** Funding acquisition (equal); investigation (equal); writing‐original draft (supporting). **Wei Liu:** Formal analysis (equal); supervision (equal); writing‐review and editing (equal). **Wen‐Jun Bu:** Supervision (equal); writing‐review and editing (equal). **Xin‐Hua Wang:** Supervision (equal); writing‐original draft (equal). **Lidong Mo:** Formal analysis (equal); software (equal); writing‐original draft (equal).

## Supporting information

File S1

File S2

File S3

Supplementary Material

## Data Availability

A list of all species, specimens, their individual images, georeferences, primers, sequences and other relevant laboratory data of all 434 specimens are available through the dataset “Global DNA barcodes of the genus *Rheocricotopus* (DS‐2020RHEO)” on the Barcode of Life Data System (http://www.boldsystems.org, BOLD), DOI: https://doi.org/10.5883/DS‐2020RHEO. Sampling locations and ecological analysis are available at the Zenodo, DOI: https://doi.org/10.5281/zenodo.5070260.
