# Supplementary figures and images for: Toward a global DNA barcode reference library of the intolerant nonbiting midge genus Rheocricotopus Brundin, 1956
Source: Ecol Evol. 2021 Aug 4;11(17):12161–72. doi: 10.1002/ece3.7979 (PMC8427567; doi:10.1002/ece3.7979)

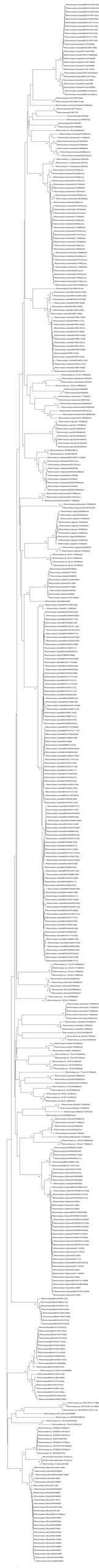

Supplement: Supplementary file 1 — File S1 [file ECE3-11-12161-s004.pdf]

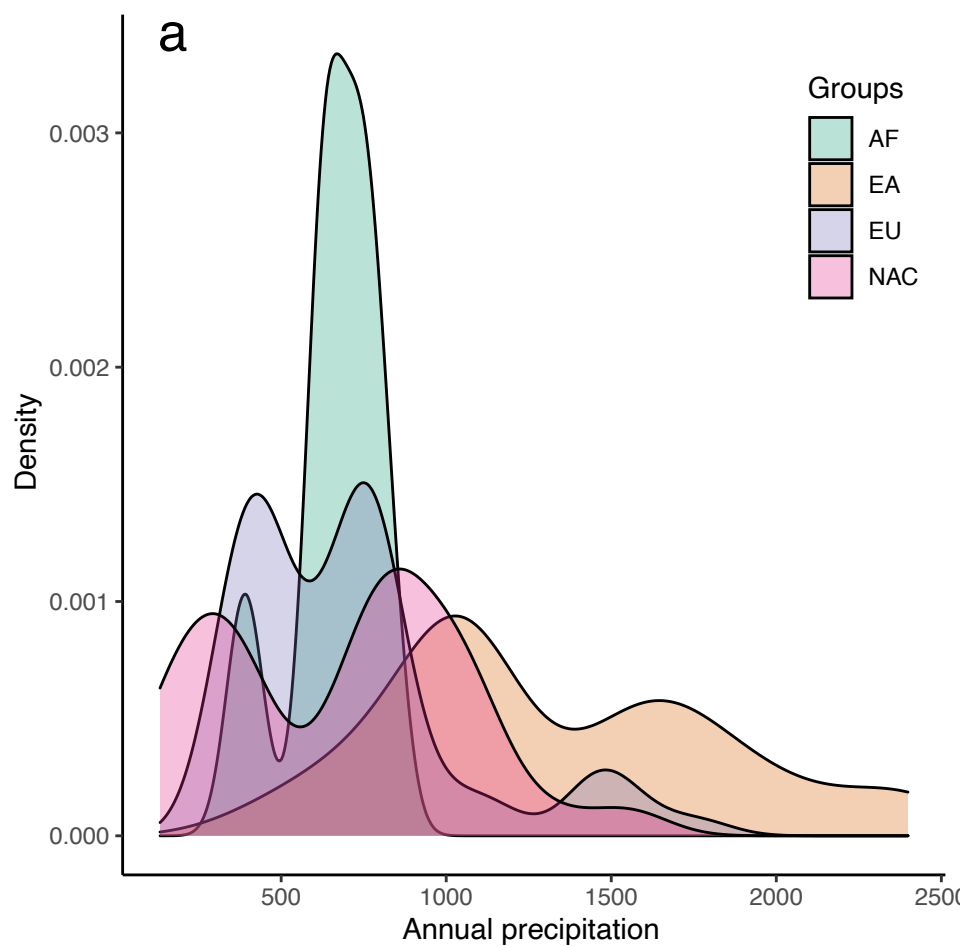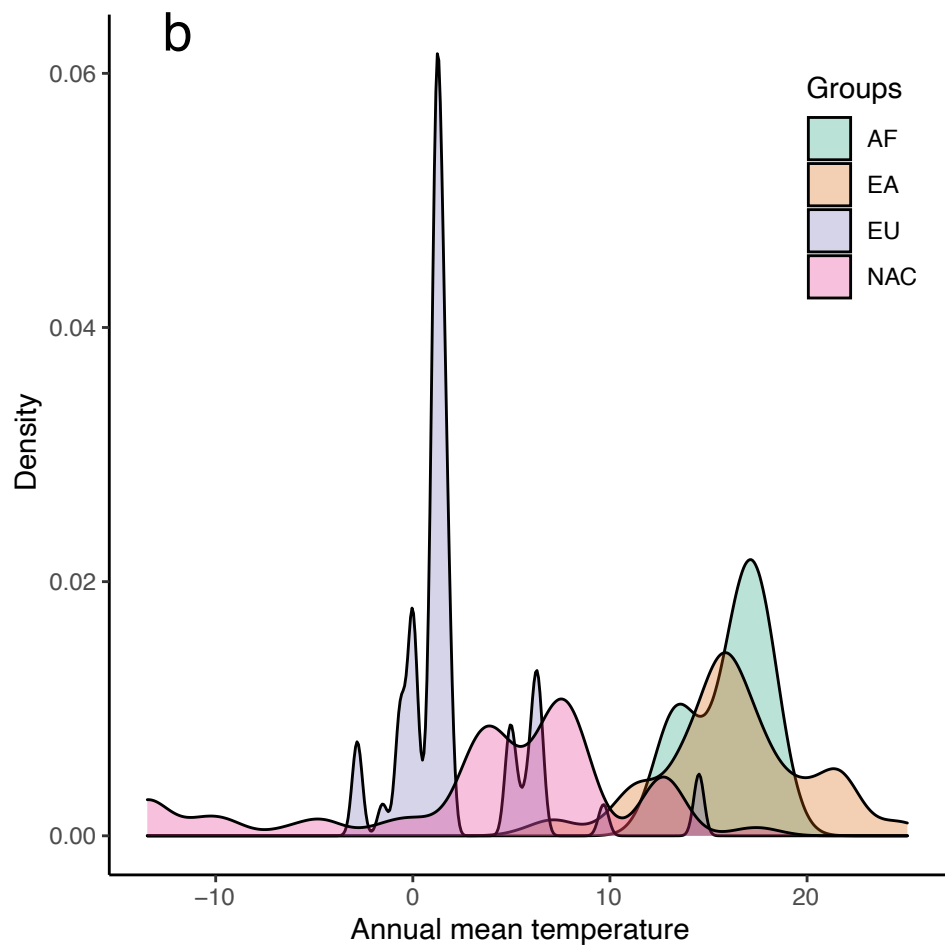

Supplement: Supplementary file 3 — File S3 [file ECE3-11-12161-s003.pdf]
