# Supplementary material for: Toward a global DNA barcode reference library of the intolerant nonbiting midge genus Rheocricotopus Brundin, 1956
Source: Ecol Evol. 2021 Aug 4;11(17):12161–72. doi: 10.1002/ece3.7979 (PMC8427567; doi:10.1002/ece3.7979)
Supplement: Supplementary file 2 — File S2 [file ECE3-11-12161-s002.pdf]

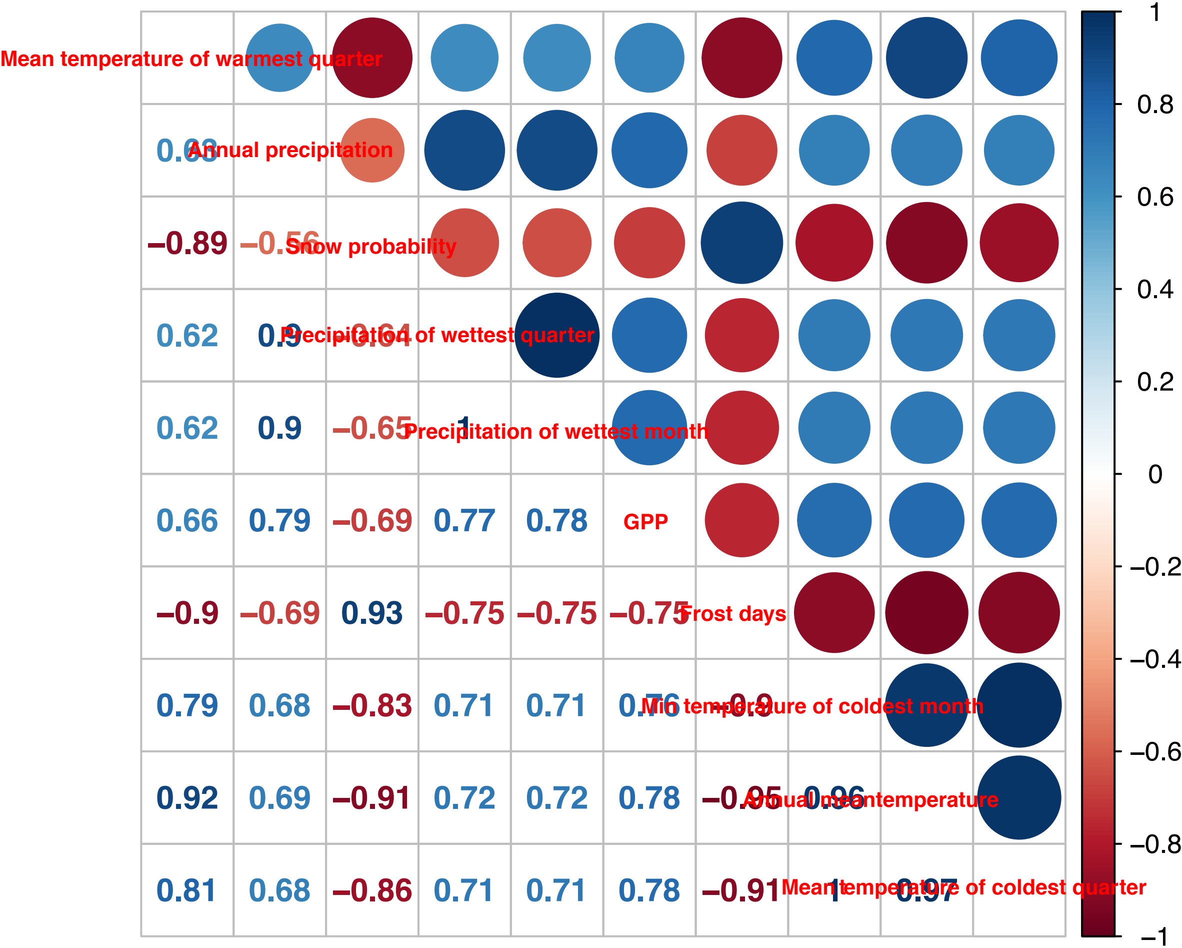
Mean temperature of warmest quarter
Annual precipitation
Snow probability
Precipitation of wettest quarter
Precipitation of wettest month
GPP
Frost days
Min temperature of coldest month
Annual meantemperature
Mean temperature of coldest quarter
−0.89
0.62
0.62 0.9 −0.65
0.66 0.79 −0.69 0.77 0.78
−0.9 −0.69 0.93 −0.75 −0.75 −0.75
0.79 0.68 −0.83 0.71 0.71
0.92 0.69 −0.91 0.72 0.72 0.78
0.81 0.68 −0.86 0.71 0.71 0.78 −0.91
1
0.8
0.6
0.4
0.2
0
−0.2
−0.4
−0.6
−0.8
−1
