# Supplementary material for: Toward a global DNA barcode reference library of the intolerant nonbiting midge genus Rheocricotopus Brundin, 1956
Source: Ecol Evol. 2021 Aug 4;11(17):12161–72. doi: 10.1002/ece3.7979 (PMC8427567; doi:10.1002/ece3.7979)
Supplement: Supplementary file 4 — Supplementary Material [file ECE3-11-12161-s001.docx]

**File S1.** Neighbor joining tree for *Rheocricotopus* species based on 434 DNA barcodes. Labels represent taxa name, BOLD Sample ID, gender and life stages. Numbers on branches represent bootstrap support (≥70) based on 1,000 replicates; scale equals K2P genetic distance.

**File S2.** Pearson’s correlation of the top 10 important variables in PCA analysis.

**File S3.** Density distribution of annual precipitation (a) and annual mean temperature (b) for the four species groups from *Rheocricotopus*. The four groups are east and southeast Asia (EA), Europe(EU), North America (NAC) and Africa (AF).
